# Design of novel JAK3 Inhibitors towards Rheumatoid Arthritis using molecular docking analysis

**DOI:** 10.6026/97320630015068

**Published:** 2019-02-28

**Authors:** Divya Jain, Trishang Udhwani, Shreshtha Sharma, Aishwarya Gandhe, Palugulla Bhaskar Reddy, Anuraj Nayarisseri, Sanjeev Kumar Singh

**Affiliations:** 1 In silico Research Laboratory,Eminent Biosciences,Mahalakshmi Nagar,Indore-452010,Madhya Pradesh,India; 2 Bioinformatics Research Laboratory,LeGene Biosciences Pvt Ltd., Mahalakshmi Nagar,Indore-452010, Madhya Pradesh,India; 3 Department of Biotechnology and Microbiology,Government PG Arts and Science College, Ratlam-457001, Madhya Pradesh,India; 4 Computer Aided Drug Designing and Molecular Modeling Lab,Department of Bioinformatics, Alagappa University,Karaikudi-630 003,Tamil Nadu,India

**Keywords:** Rheumatoid Arthritis, JAK 3 inhibitor, Molecular docking, Virtual screening, BOILED-Egg plot, ADMET

## Abstract

Multiple cytokines play a pivotal role in the pathogenesis of Rheumatoid Arthritis by inducing intracellular signaling and it is known that the members of the Janus kinase (JAK) family are essential for such signal transduction. Janus kinase 3 is a tyrosine kinase that belongs to the Janus family of kinases. Drugs targeting JAK3 in the treatment of Rheumatoid arthritis is relevant. Therefore, it is of interest to design suitable inhibitors for JAK3 dimer using molecular docking with Molegro Virtual Docker. The compound possessing the highest affinity score is subjected to virtual screening to retrieve inhibitors. The compound SCHEMBL19100243 (PubChem CID- 76749591) displays a high affinity with the target protein. The affinity scores of this compound are more than known drugs. ADMET analysis and BOILED Egg plot provide insights into this compound as a potent inhibitor of JAK3.

## Background

Rheumatoid arthritis (RA) is defined as a chronic inflammatory disorder that primarily affects joints but can spread to other body systems, including the skin, eyes, lungs, heart and blood vessels. An autoimmune disorder, rheumatoid arthritis occurs when your immune system mistakenly attacks self-tissues and starts attacking the lining of your joints, causing a painful swelling that can eventually result in bone erosion and joint deformity. The multiple cytokines play pivotal roles in RA pathogenesis by inducing intracellular signaling, and members of the Janus kinase (JAK) family are essential for such signal transduction [1]. JAK3 is an intracytoplasmic tyrosine kinase that is physically and practically coupled to gamma chain permitting cytokine subordinate flag transduction. Janus Kinase (JAKs) assumes a fundamental job in cytokine receptor motioning since they phosphorylate and enact flag transducer and activator of translation (STAT) protein. A few of these JAK controlled cytokine receptor pathways are personally engaged with the intention and movement of Rheumatoid Arthritis sickness pathogenesis. The JAK/STAT pathway is generally communicated intracellular flag transduction pathway, on a very basic level essential for T lymphocyte separation and capacity.

Selective inhibition of JAK3 has been identified as an important strategy for the treatment of autoimmune disorders [3]. Based on the unique Cys909 of JAK3 at the gatekeeper position, a new irreversible covalent inhibitor (III-4) which is highly potent and selective in targeting JAK3 [2]. Tofacitinib is a disease-modifying antirheumatic drug (DMARD) which was recently approved by the US Food and Drug Administration (FDA). There are several randomized clinical trials (RCTs) that have investigated the efficacy and safety of tofacitinib in adult patients with rheumatoid arthritis (RA). A systematic review with a meta-analysis of RCTs was undertaken to determine the efficacy and safety of tofacitinib in treating patients with RA [3]. The efficacy, safety and dose response of a oral Janus kinase inhibitor named peficitinib (ASP015K) as a mono therapy in Japanese patients with moderate to severe rheumatoid arthritis (RA). Peficitinib 50, 100 and 150 mg each showed statistically significantly higher ACR20 response rates compared with placebo, and response rates increased up to 150 mg with a statistically significant dose-response is known [4]. Decernotinib (VX-509), an oral selective inhibitor of JAK-3, was also tested in patients with rheumatoid arthritis (RA) in whom the response to methotrexate treatment was inadequate. VX-509 significantly improved the signs and symptoms of RA at weeks 12 and 24 compared with the placebo group when it was administered in combination with methotrexate [5]. Moreover, (JAK3) is expressed in lymphoid cells and is involved in the signaling of T cell functions. The development of a selective JAK3 inhibitor has been shown to have a potential benefit in the treatment of autoimmune disorders [6]. PF-06651600, a newly discovered potent JAK3-selective inhibitor, is highly efficacious at inhibiting γc cytokine signaling, which is dependent on both JAK1 and JAK3. PF-06651600 allowed the comparison of JAK3-selective inhibition to pan-JAK or JAK1-selective inhibition, in relevant immune cells to a level that could not be achieved previously without such potency and selectivity [7]. Therefore, it is of interest to design inhibitors against JAK3 dimeric structure using molecular docking and virtual screening.

## Methodology

### Selection of JAK3 inhibitors:

**Figure 1:**
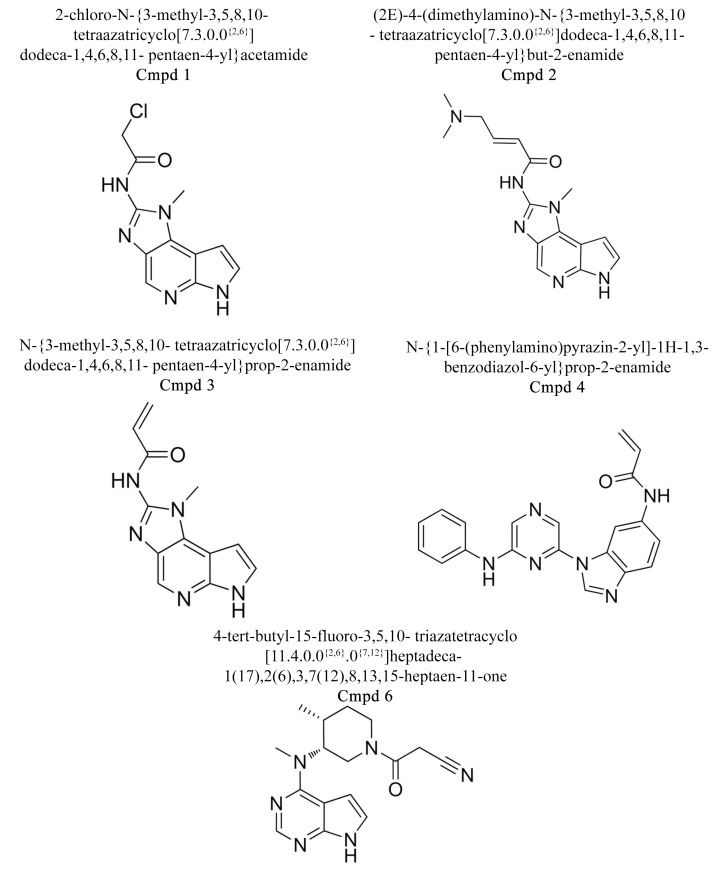
Established Inhibitors of JAK3 without PubChem ID [20].

Literature findings were conducted to find pre-established inhibitors of JAK-3 which were adept to binding and hence for restraining the activity of the protein. The aggregate number of established inhibitors was found to be 17, which were chosen for further analysis. The structures of 12 were available in the PubChem database from which these were directly downloaded while the 3D structures (Table 1) of remaining compounds were built using MarvinSketch and were saved in the 3D.sdf format (Figure 1).

### Protein and ligand preparation:

**Figure 2:**
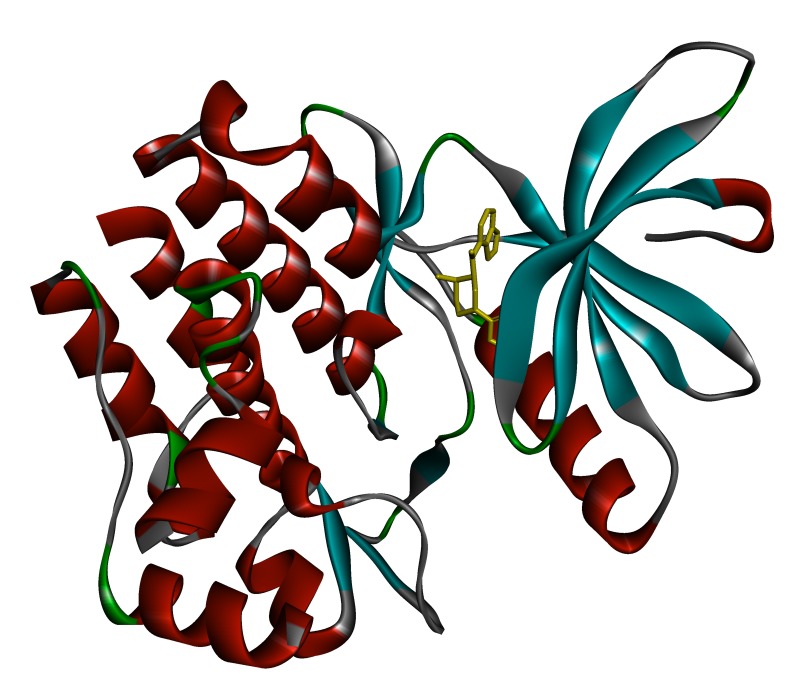
Protein 3D structure of JAK3 obtained from PDB (PDB ID: 3LXK)

The crystal structure of the target protein JAK3 was obtained from Protein Data Bank (PDB) with PBD ID: 3LXK [21] as shown in Figure 2. Ligand preparation was carried out by taking the 3D structures of retrieved as well as constructed ligands and processing them using the LigPrep module of Schrodinger suite, 2013 (Schrodinger. LLC, New York, NY) where, these were optimized through OPLS 2005 force field algorithm [22-26]. This preparation resulted in all the ligand structures in a single file, which was saved with a .sdf extension for docking with the target protein [27-29].

### Molecular docking:

Using Molegro Virtual Docker (MVD), which unified high potential Piece-Wise Linear Potential (PLP) and MolDock scoring function [30-33], molecular docking analyses were carried out. The protein was first loaded in the Docker where it was prepared by removing the pre-existing ligand from the protein structure [34-36]. Cavity one was witnessed to possess the largest volume and the ligand structure docked within it and was thence utilized for docking of the prepared ligands [37-41]. The single .sdf file created in the previous step was taken for loading all the ligand structures in the docker. Docking procedure-holding parameter of maximum iteration of 1500, grid solution 0.2 having a binding affinity, maximum population size 50, the protein and ligands were assessed on the subsequent confirmation of the Internal Electrostatic interaction (Internal ES), sp2-sp2 torsions, and internal hydrogen bond interaction [42-46]. Energy minimizationand Hbond optimization were carried out after docking. Placing of Simplex Evolution at max steps 300 and neighbor distance faster 1.00. After docking to minimize the complex energy of ligandreceptor interaction the Nelder Mead Simplex Minimization (using non- grid force field and H-bond directionality) was used [47-52].

### Virtual screening:

The compound, which showed the highest re-rank score value in the docking table was considered as the best-established drug. Similarity search was carried out against this best-established compound to get a superior compound possessing a larger binding affinity to the 3D crystal structure, other than any previously established drugs [53-57]. This similarity searching was carried out against PubChem database developed by NIH, one of the public chemical repositories, which contain structures of 93 million chemical compounds [58-61]. The filtration property parameter set by component rule of Lipinski's rule of five was set at threshold >=95. These compounds were downloaded in sdf format and docked using the identical procedure with the crystal structure of JAK3 protein to find the compound showing a higher affinity towards the target protein than the best-established drug.

### Drug-Drug Comparative Study:

Docking of established drugs with the help of Molegro Virtual Docker led to the creation of a docking folder. An 'unnamed complex' structure file was created in this folder. This structure file was opened with the help of Molegro and all constraints, cavities, and ligands in the structure were removed to obtain only the protein structure [62-63]. The best pose of the drug was tallied from the result generated and was then imported. The resultant structure generated was saved as the best-posed drug and was stored in PDB format. Similarly, the 'unnamed complex' structure file resulting from the docking of virtually screened compounds was retrieved from its respective folder and the steps were reiterated to obtain the best virtually screened drug pose. An excel sheet was organized to check and compare all the affinities, hydrogen interaction, steric energy and high re-rank score to draw out a comparison between the two drugs [40,44].

### ADMET studies:

The admetSAR database provides a free and open web resource, which gives an estimation of the biological and chemical profile of the compound entered. The resource is available at http://lmmd.ecust.edu.cn:8000/. Properties stated in the ADMET profile include digestion, adsorption, metabolism, toxicity, excretion and so on. These give us in-depth information regarding the development and discovery of drug in question. The database is divided into 22 qualitative classifications and 5 quantitative regression models, which aim to provide a comprehensive outcome with high precision based on estimation. Hence, this database was used to estimate the properties of the inhibitors under study. The analysis was made for the best- established compound to facitinib and the best virtual screened compound with PubChem CID: 76749591 to predict the bioactivity properties and toxicity using admetSAR [44].

### Boiled-egg plot

A BOILED-Egg plot lends reassuring assistance and provides a unique statistical plot to support the two passive predictions made, which is gastrointestinal absorption and brain penetration of small molecules, which is essential for discovery, and development of drugs. Both the parameters are represented on a cartesian plane in the shape of eclipses and include other important parameters such as MW, TPSA, MLOGP, GI, and BBB to recondition the BOILEDEgg plot. Accordingly, in the cartesian plane, if our compounds rest in the yolk region represented by the yellow ellipse, the probability of BBB (Blood Brain Barrier) is escalated whereas if the compounds rest under white areas, the conjecture of gastrointestinal absorption is amplified. Beside these regions, if the compounds rest in gray areas excluding the 'egg' or are out of range of the graph, the compounds are non-absorptive even non-brain penetration and hence it contemplated as a remarked box. The regions are not exclusive of each other [40,44,48].

### Software, Suites and Web servers Used:

Retrieval of inhibitor structures was done from NCBI's PubChem database in 3DSDF format. The inhibitors which lacked PubChem CID or the 3D structure was absent in PubChem were drawn using MarvinSketch5.6.0.2, (1998-2011, Copyright ChemAxonLtd). Ligand optimization was done using Schrodinger suite (Schrodinger, LLC, 2009, New York, NY). Molegro Virtual Docker 2010.4.0.0 was used for flexible docking of receptor protein structure and all ligand structures. Molecular Visualization was conducted with the support of Accelrys Discovery Studio^®^ Visualizer 3.5.0.12158 (Copyright^©^ 2005-12, Accelrys Software Inc.). ADMET profiles were predicted and organized using admetSAR (Laboratory of Molecular Modeling and Design. Copyright (2012) East China University of Science and Technology, Shanghai Key Laboratory for New Drug-Drug Design).

## Results and discussion:

The docking results of all pre-established drugs, when docked in the cavity 1 of the JAK3 protein structure show that Tofacitinib (CP 690,550) represented in the table as Cmpd5 displays the best interaction (Table 2). Some of the properties of this compound include a molecular weight of 312.37 and a measured logP value of 1.24. The compound has 1 hydrogen bond donor and 5 hydrogen bond acceptors. The IUPAC name of the compound is 3-[(3R,4R)-4- methyl-3-[methyl(7H-pyrrolo[2,3-d]pyrimidin-4- yl)amino]piperidin-1-yl]-3-oxopropanenitrile.

## Virtual Screening Results:

Similarity searching for this inhibitor against the PubChem database resulted in 314 compounds, which show a very similar structure to the best-established drug. Table 3 lists the top 10 docking results of these virtually screened compounds. The table establishes compound SCHEMBL19100243 (PubChem CID-76749591) as the best virtual docked compound. The compound displays physical properties such as a molecular weight of 380.444g/mol, 3 hydrogen bond donors, and 5 hydrogen bond acceptors. It is also clear from the table that the re-rank score of this compound (-134.539) is lower than the re-rank score of the bestestablished drug that is CP690, 550 which indicates its greater affinity to the target protein.

Table 4 compares the interaction energies of the best-established compound CP 690,550 (tofacitinib) with the best virtual screened compound PubChem CID: 76749591. The re-rank scores of both the compounds show that the virtual screened compound binds with far more affinity to JAK 3 receptor when compared to the bestestablished drug. The MolDock scores of these drugs show an even more superiority of the virtual screened drug. The same trend is mimicked in all the descriptors, with the virtual screened drug surpassing the established drug by large margins. External Ligand interactions, protein-ligand interactions, and steric interactions replicate these results. The hydrogen bond energies of both the compounds are relatively the same. Based on this table it can be concluded that the best virtual screened drug has the potential to bind with greater affinity and can hence be used with a superior effect in the treatment of Rheumatoid Arthritis.

**Figure 3:**
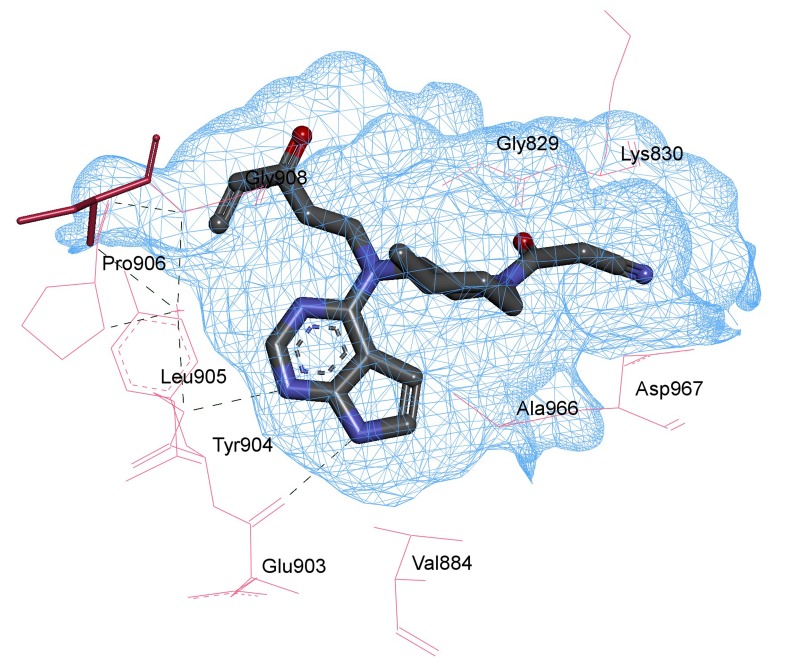
The compoundSCHEMBL19100243 (PubChem CID- 76749591), the most effective virtual screened drug shows ligand-receptor interactions.

**Figure 4:**
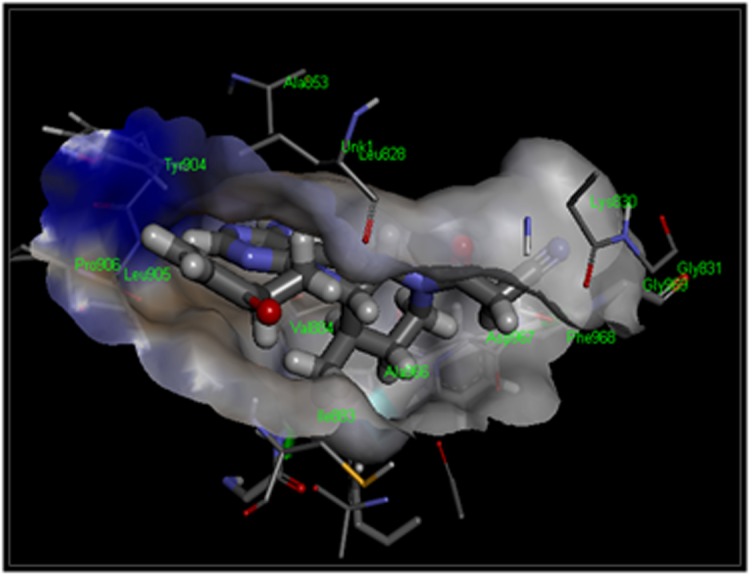
The compoundSCHEMBL19100243 (PubChem CID- 76749591), the most effective virtual screened drug shows aromatic interactions.

Pharmacophore mapping provides us with tools for spatial systematic topographies of molecular interaction with a specific target protein receptor and serves as an alternative to the procedure of molecular docking. These studies help convey a precise query on the finest interface of the inhibitor with its target protein, aided by annotations and represent the aligned poses of the molecule and help to search for high interactions between the target protein and the inhibitor under study. The interaction of the receptor protein JAK3 is found to be quite effective with the drug SCHEMBL19100243 (PubChem CID - 76749591), pharmacophore studies are held to further understand different interactions that are present in the complex so formed. The interactions carried out for the purpose of this study include hydrogen bond interactions, van der walls interaction, aromatic interactions and ligand interactions. Figure 3 displays receptor-ligand interaction shown by the virtual screened compound SCHEMBL19100243 (PubChem CID- 76749591 in the cavity of JAK-3 protein structure. Primary interaction between Leu 905 and the N5 of the ligand and Glu 903 and N4 of the ligand can be seen to provide affinity to keep the structure intact. Figure 4 highlights the best-virtual screened compound SCHEMBL19100243 (PubChem CID- 76749591) showing aromatic interaction in the binding cavity of protein JAK3. The protein cavity can be seen to be shaded in two different colors, with surfaces portraying blue color signifying the edges and while the shade surfaces displaying dull orange color signifying the face.

**Figure 5:**
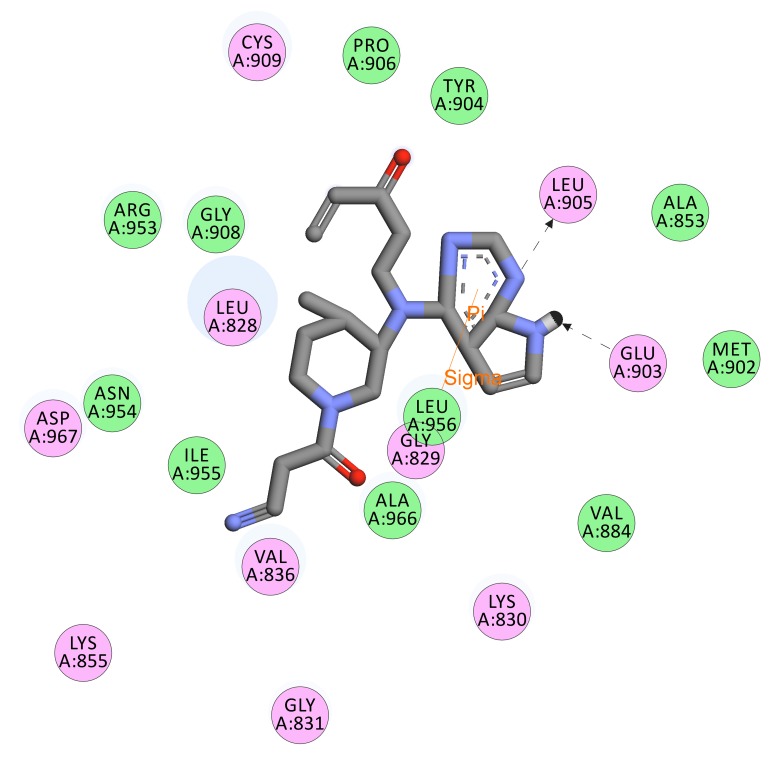
The compound SCHEMBL19100243 (PubChem CID- 76749591), the most effective virtual screened drug shows van der walls interactions.

Figure 5 presents the interacting residues of the JAK3 protein structure with the inhibitor SCHEMBL19100243 (PubChem CID- 76749591) embedded in its cavity. The residues in pink circles display electrostatic interactions whereas those in green represent van der walls interactions. Green dotted arrows between the interacting species denote hydrogen bonds. Hence, it can be concluded that Glu 903 acts as a hydrogen bond donor whereas Leu 905 acts as a hydrogen bond acceptor. Also, there is a formation of a sigma- pi bond between the inhibitor and Leu 956. Additionally, it can be observed that residues Pro 906, Tyr 904, Ala 853, Met 902, Val 884, Leu 956, Ala 966, Ile 955, Asn 954, Arg 953, Gly 908 show van der walls interaction with the high-affinity drug.

Table 5summarizes the ADMET prediction of both the bestdocked compound Tofacitinib (CP 690,550) andPubChem CID 76749591. It can be seen that the BBB (Blood Brain Barrier) values of both these compounds are almost equivalent, while the virtual screened compound shows better value for Human Intestinal Absorption (HIA), which is the prediction of absorption of the drug in the intestine. All other absorption criteria favor the virtual screened drug as better figures are presented in that column. Metabolism criteria of both these compounds are again almost equivalent, with some properties favoring the best-virtual screened drug. Both these compounds are non-carcinogens. When comparing the toxicity criteria, it can again be said that the virtual screened drug edges over the best-established drug. Both these compounds are also shown to be not easily biodegradable. Table 6 summarizes the comparison of the regression prediction of ADMET analysis of the two drugs under consideration. The regression model shows that the virtual screened drug has a higher CaCo2 permeability in regression studies. Toxicity studies the virtual screened drug shows lower levels of rat acute toxicity as well as fish toxicity when compared to the best-established drug.

**Figure 6:**
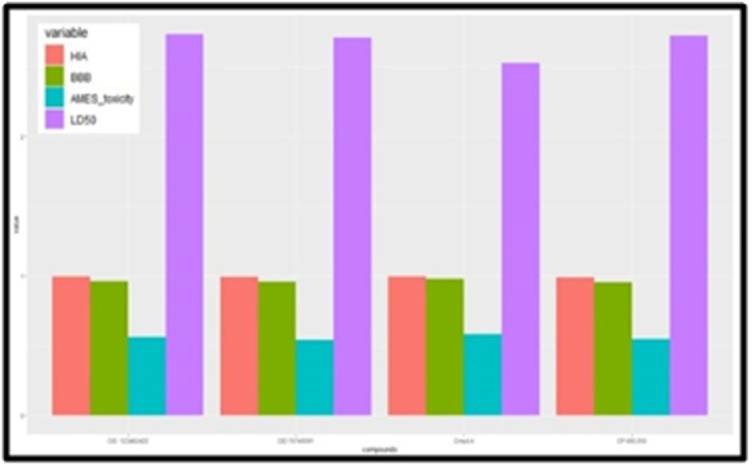
Comparative ADMET studies of BBB, HIA, AMES toxicity and LD50 of the Established compounds against Virtual screened compounds. Expand abbreviations used in this Figure

A relative ADMET profile comparison was carried out for selected inhibitors. Predictions were based on parameters such as the Blood- Brain Barrier (BBB), Human Intestinal Absorption (HIA), AMES Toxicity, and LD50 rat toxicity. The established inhibitorCP 690,550 (Tofacitinib) and Cmpd4, the virtual screened drugs PubChem CID 76749591 and PubChem CID 123462422 were taken up for comparison according to ADMET studies. These four inhibitors were graphically represented using R-programming as highlighted in  Figure 6 and Table 7. The parameters, BBB, HIA, AMES Toxicity, and LD50 acquired from the admetSAR database and were tabulated according to their estimated values. The best virtual screened compound PubChem CID 7674959 is seen to have the lowest AMES toxicity levels in mice among all the drugs. Also, this inhibitor shows the lowest levels of the Blood-Brain Barrier (BBB).The virtual screened compound shows Human Intestinalabsorption values more than that compared to the best-established drug CP 690,550.

**Figure 7:**
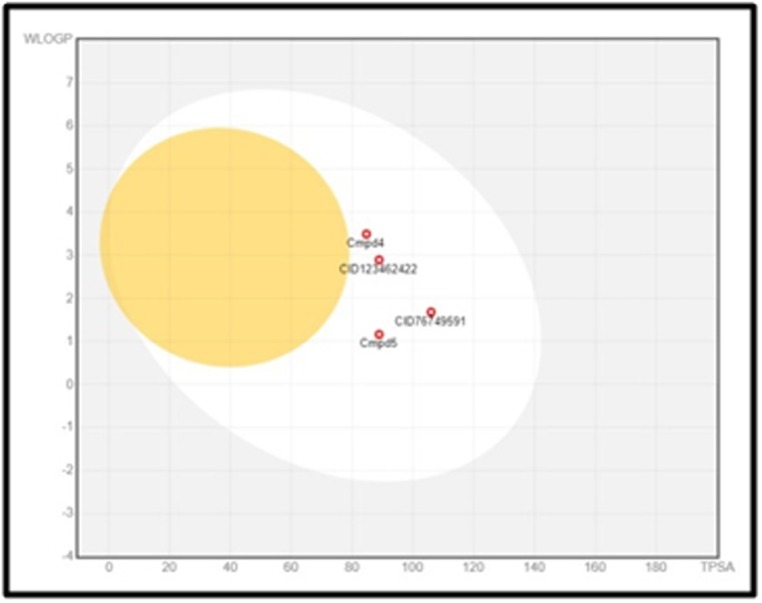
Boiled-egg Plot

The compounds: CP 690,550 (Tofacitinib) and Cmpd 4, and the top two virtually screened compounds (PubChem CID76749591 and PubChem CID123462422) were plotted in the BOILED -Egg plot. Table 8 summarizes the results of the plot. Observations indicate that all four drugs show high GI absorption and a negative result for Blood-Brain permeation. This observation justifies the placement of all the four compounds in the white region of the BOILED- Egg plot. The virtual screened drug with PubChemCID76749591 shows the highest value for TPSA and lies almost in the center of the white region. None of the compounds fall in the grey region of the plot, which confirms that all these compounds display high GI absorption and are all BBB permeable (Figure 7).

## Conclusion

The known drugCP690,550 (Tofacitinib) shows a high degree of binding to the JAK 3 receptor. We describe a compound SCHEMBL19100243 (PubChem CID-76749591) that surpasses the affinity scores of CP690,550. The drug-drug comparison scores highlight the supremacy of this drug over all the previously established drugs, evident by comparing the re-rank scores. The pharmacophore mapping of the molecule shows the efficiency with which it binds to the receptor structure. The ADMET profile of this ligand is highly favorable, which predicts the ligand would give positive results when in vitro and in vivo studies are conducted. Furthermore, the boiled-egg plot confirms the ADMET results, adding weight to the potential for the virtual-screened ligand as a JAK3 inhibitor towards rheumatoid arthritis.

## Figures and Tables

**Table 1:** Established Inhibitors of JAK3 with PubChem ID (if structures are present in PubChem) with properties

SNo	Inhibitor	Pub Id	M. W	HBA	HBD	Ref
1	tofacitinib	9926791	312.377 g/mol	1	5	[8-10]
2	peficitinib (ASP015K)	57928403	326.4 g/mol	4	4	[11-13]
3	Decernotinib (VX-509)	59422203	392.386 g/mol	3	8	[14,15]
4	RB1	9602155	271.32	2	3	[16,17]
5	Oxindole inhibitor	321710	133.15 g/mol	1	1	[18]
6	PF-06651600	118115473	285.351 g/mol	2	4	[7,19]
7	Tricyclic 1		263.38	2	3	[19]
8	Tricyclic2	4592	298.34	2	4	[19]
9	Tricyclic3	5325595	241.25	2	3	[19]
10	Tricyclic4		356.38	2	4	[19]
11	Tricyclic5	25180101	312.37	1	4	[19]
12	Tricyclic6	5494425	309.34	2	3	[19]

**Table 2:** Established drug docking result

Name	Ligand	MolDock Score	Rerank Score	Interaction	H-Bond	MW
[00] Cmpd5	Cmpd5	-139.109	-118.575	-150.279	-5.00779	312.37
[01] Cmpd4	Cmpd4	-137.471	-116.71	-157.659	-1.8916	356.381
[00] 9926791	9926791	-132.532	-115.618	-149.689	-5.01012	312.37
[02] Cmpd4_1	Cmpd4_1	-134.428	-114.697	-157.149	-2.44973	357.389
[01] 59422203	59422203	-144.113	-112.997	-157.552	-6.40297	392.378
[00] 59422203	59422203	-138.499	-112.487	-157.141	-0.05029	392.378
[00] Cmpd4	Cmpd4	-141.412	-110.582	-166.422	-1.64215	356.381
[02] Cmpd5	Cmpd5	-128.311	-109.219	-141.045	-2.58482	312.37
[00] Cmpd6_2	Cmpd6_2	-129.514	-109.036	-138.288	-5.49497	310.345
[00] Cmpd6_1	Cmpd6_1	-129.111	-109.026	-138.179	-5	309.338

**Table 3:** Virtual screened drug docking result

Name	MolDock Score	Rerank Score	HBond	Heavy Atoms	MW
[00]76749591	-163.777	-134.539	-4.54815	28	380.444
[00]123462422	-169.302	-133.688	-4.84579	28	380.487
[00]58264150	-161.85	-132.198	-4.69423	28	380.487
[00]58263597	-160.611	-128.981	-4.99925	28	381.432
[00]58263953	-163.6	-128.677	-4.99763	27	366.46
[01]58263953	-155.397	-128.607	-4.73386	27	366.46
[00]123228386	-162.136	-128.572	-5.02263	28	381.432
[01]76749591	-159.869	-127.965	-4.79724	28	380.444
[00]59772932	-160.135	-127.099	-6.71056	28	402.43
[03]126513890	-156.203	-126.928	-3.98844	28	381.432

**Table 4:** Drug-Drug Comparative study

	Best Established compound: CP 690, 550 (Tofacitinib)		Best Virtual Screened compound: PubChem CID 76749591	
Energy overview: Descriptors	MolDock Score	Rerank Score	MolDock Score	Rerank Score
Total Energy	-139.749	-118.272	-163.788	-134.546
External Ligand interactions	-139.575	-117.041	-170.157	-146.483
Protein - Ligand interactions	-134.575	-117.041	-170.157	-146.483
Steric (by PLP)	-139.823	-95.919	-165.611	-113.609
Steric (by LJ12-6)		-17.359		-29.273
Hydrogen bonds	-4.752	-3.763	-4.546	-3.601
Internal Ligand interactions	14.826	16.77	6.369	11.937
Torsional strain	7.222	6.774	2.167	2.033
Torsional strain (sp2-sp2)		2.796		0.336
Hydrogen bonds		0		0
Steric (by PLP)	7.604	1.308	4.202	0.723
Steric (by LJ12-6)		5.891		8.846

**Table 5:** ADMET Predicted Profile and Classification

	Best Virtual Screened Drug:PubChem CID 76749591		Best Established Drug: CP 690,550 (Tofacitinib)	
Model	Result	Probability	Result	Probability
Absorption				
Blood-Brain Barrier	BBB+	0.9598	BBB+	0.9568
Human Intestinal Absorption	HIA+	0.9956	HIA+	0.9897
Caco-2 Permeability	Caco2-	0.5686	Caco2+	0.5154
P-glycoprotein Substrate	Substrate	0.6712	Substrate	0.6524
P-glycoprotein Inhibitor	Inhibitor	0.932	Inhibitor	0.7609
	Inhibitor	0.9773	Inhibitor	0.8898
Renal Organic Cation Transporter	Inhibitor	0.5956	Inhibitor	0.6368
Distribution				
Subcellular localization	Mitochondria	0.3864	Mitochondria	0.37
Metabolism				
CYP450 2C9 Substrate	Non-substrate	0.8175	Non-substrate	0.8246
CYP450 2D6 Substrate	Non-substrate	0.7281	Non-substrate	0.723
CYP450 3A4 Substrate	Substrate	0.6923	Substrate	0.7649
CYP450 1A2 Inhibitor	Non-inhibitor	0.7134	Non-inhibitor	0.734
CYP450 2C9 Inhibitor	Inhibitor	0.5072	Non-inhibitor	0.8014
CYP450 2D6 Inhibitor	Non-inhibitor	0.9081	Non-inhibitor	0.9537
CYP450 2C19 Inhibitor	Non-inhibitor	0.5384	Non-inhibitor	0.8036
CYP450 3A4 Inhibitor	Non-inhibitor	0.6549	Non-inhibitor	0.9307
CYP Inhibitory Promiscuity	High CYP Inhibitory Promiscuity	0.5527	Low CYP Inhibitory Promiscuity	0.7937
Toxicity				
Human Ether-a-go-go-Related Gene Inhibition	Strong inhibitor	0.6427	Weak inhibitor	0.5995
	Inhibitor	0.518	Inhibitor	0.7324
AMES Toxicity	Non AMES toxic	0.5407	Non AMES toxic	0.5492
Carcinogens	Non-carcinogens	0.8741	Non-carcinogens	0.9032
Fish Toxicity	High FHMT	0.9553	High FHMT	0.7677
Tetrahymena Pyriformis Toxicity	High TPT	0.9269	High TPT	0.8348
Honey Bee Toxicity	Low HBT	0.8765	Low HBT	0.8848
Biodegradation	Not ready biodegradable	0.9934	Not ready biodegradable	0.9956
Acute Oral Toxicity	III	0.6154	III	0.6845
Carcinogenicity (Three-class)	Non-required	0.5991	Non-required	0.6912

**Table 6:** ADMET Predicted Profile and Regression

	Best Virtual Screened Drug PubChem CID 76749591		Best Established Drug CP 690,550	
Model	Value	Unit	Value	Unit
Absorption				
Aqueous solubility	-3.6174	LogS	-2.9488	LogS
Caco-2 Permeability	0.8086	LogPapp, cm/s	0.5977	LogPapp, cm/s
Toxicity				
Rat Acute Toxicity	2.7101	LD50, mol/kg	2.7249	LD50, mol/kg
Fish Toxicity	1.1207	pLC50, mg/L	1.3125	pLC50, mg/L
Tetrahymena Pyriformis Toxicity	0.562	pIGC50, ug/L	0.5293	pIGC50, ug/L

**Table 7:** Comparative ADMET profile of the test ligands and the control

	Blood-Brain Barrier	Human Intestinal Absorption	AMES Toxicity	Carcinogenicity	LD50 in rats
CP 690,550 (Tofacitinib)	0.9568	0.9897	0.5492	Non- carcinogenic	2.7249
Cmpd 4	0.9806	0.9958	0.584	Non- carcinogenic	2.5278
PubChem CID 76749591	0.9598	0.9956	0.5407	Non- carcinogenic	2.7101
PubChem CID 123462422	0.9631	0.9973	0.5608	Non- carcinogenic	2.7365

**Table 8:** Boiled egg parameters

Molecule	MW	TPSA	XLOGP3	MLOGP	GI absorption	BBB permeant
Cmpd5	312.37	88.91	1.5	0.7	High	No
Cmpd4	356.38	84.73	2.93	2.01	High	No
CID76749591	380.44	105.98	1.74	0.7	High	No
CID123462422	380.49	88.91	3.19	1.79	High	No
